# Metricizing policy texts: Comprehensive dataset on China’s Agri-policy intensity spanning 1982–2023

**DOI:** 10.1038/s41597-024-03367-0

**Published:** 2024-05-22

**Authors:** Yehui Wu, Canyu Wang, Rongbiao Ji, Yadong Li, Junkui Yang, Yixuan Wang, Rujia Li, Mengyao Wu, Jiaojiao Chen, Jianping Yang

**Affiliations:** 1 College of Big Data, Yunnan Agricultural University, Kunming, 650201 Yunnan China; 2 YuXi Agriculture Vocation-Technical College, Yuxi, 653106 Yunnan China; 3 College of Resources and Environmental Sciences, Nanjing Agricultural University, Nanjing, 210095 Jiangsu China

**Keywords:** Agriculture, Developing world

## Abstract

Due to the lack of direct assessment metrics, existing studies on the intensity of agricultural policies often utilize indicators such as Gross Domestic Product (GDP) of agriculture or the quantity of agricultural policies as measures. Optimizing methods for analyzing the intensity of agricultural policies will significantly impact parameter selection in agricultural policy research and the evaluation of policy effectiveness. In this study, we constructed a Chinese Agricultural Policy Corpus using agricultural policies released by various governmental agencies at the national level in China from 1982 to April 2023. We quantified the values of agricultural domain terms in the corpus and evaluated the intensity of each agricultural policy document. The validation results of this study indicate a strong correlation between the intensity of agricultural policies and agricultural GDP. The trend in agricultural GDP changes lags behind policy intensity by 2.5 years (at a 95% confidence level), thus validating the rationality of our constructed corpus, agricultural policy scoring dataset, and methodology.

## Background & Summary

In recent decades, the field of agriculture has undergone continuous progress and innovation to meet the increasing global demand for food, maintaining the stability and sustainability of food supply has become one of the significant challenges faced by global society^1^. Following rapid socio-economic transformation and the implementation of family planning policies in China, a trend of population aging has emerged. Despite ongoing changes in Chinese agriculture, small-scale farming remains a major component^2^. According to official data from the China State Council Census Office in 2022, only 19% of the agricultural labor force in China was below 40 years old in 2020. Many rural areas heavily rely on elderly individuals aged 60 and above for agricultural labor. With China being the most populous developing country globally, where approximately 35% of the population is engaged in agricultural labor (a proportion significantly higher than the 2.5% in the United States), the importance of agriculture to China is self-evident^3^. In recent years, China has made significant strides in the field of agriculture, thanks to the implementation of a series of agricultural policies that play a crucial role in the country’s economic and social development.

Quantitative analysis of the scale of agricultural policies directly influences the assessment of policy outcomes, providing a method for monitoring policy effects and improving the agricultural policy system. Since 2015, the Chinese government has introduced the Precision Poverty Alleviation (TPA) plan, aiming to elevate the living standards of the entire population above the national poverty line by 2020^4^. Given the substantial number of agricultural laborers in China, future agricultural policy formulation will face increasingly diverse demands, resulting in the growing complexity of policy development^5,6^. With the widespread adoption of big data and artificial intelligence, there is an urgent need to research an effective process and methods for quantifying agricultural policies.

In response to policy changes^7,8^, the outcomes of policy announcements^9,10^, and discussions regarding governmental involvement in policy formulation^11^, numerous scholars have shown considerable interest in policy documents. In recent years, an increasing body of research advocates for the use of policy outcome data to assess their impacts through more intuitive methods and to measure policy effects and outcomes more accurately^12^. Scholars such as Chen Mei *et al*.^13^ have found that with the digitization process, policy texts are rapidly increasing, making the evaluation of their design rationality and potential optimization space critical. Quantitative assessment of policies has become an indispensable scientific tool, allowing for a comprehensive and impartial examination of existing policies and guiding future improvement directions. Sun Yan *et al*.^14^, by utilizing a decade’s worth of Chinese modern agricultural policy texts, employed a classification method to construct an analytical framework and conducted quantitative analysis, deepening the understanding of policy essence. It is hoped that through quantitative analysis of agricultural policy texts, policies can be refined and made more efficient, driving the modernization of agriculture. However, current research on the overall quantitative assessment of policy data remains insufficient. Therefore, there is an urgent need to broaden the research horizon, strengthen the application and study of quantitative evaluation methods for policies to meet the constantly increasing demands of data governance. Furthermore, there is no unified measurement standard when systematically evaluating the intensity of agricultural policies across different spatial and temporal contexts^15^. Based on the current state of knowledge, there has yet to be a comprehensive systematic study capable of fully collecting and publicly disclosing Chinese agricultural policy data.

To address the information and methodological gaps in this field, this study comprehensively collects relevant documents on important agricultural policies at the national level in China, constructing an agricultural policy corpus. We introduce for the first time the temporal dimension and clustering algorithm DBSCAN on the basis of the LDA (Latent Dirichlet Allocation) model to partition the topics. By leveraging the new machine learning model EvoLDA-DB (Evolutionary LDA with DBSCAN), we construct a comprehensive dataset of agricultural policy corpus to achieve a quantitative assessment of the intensity of agricultural policies. The dataset comprises the agricultural policy corpus at the national level in China over the past 40 years. We propose a novel method to obtain quantified datasets of agricultural policy corpus words and scores for each document. This approach to computing policy intensity can be applied not only for inter-country comparisons but also across different domains. Building upon the multiclustered partition of corpus words provided by this study, it offers more practical support for further analysis of different aspects or dimensions within this field. Furthermore, the dataset of this study provides detailed content of Chinese agricultural policy documents categorized by different government departments, aiding researchers in reducing the time and effort required to access information on Chinese agricultural policies.

## Methods

**Fig. 1:**
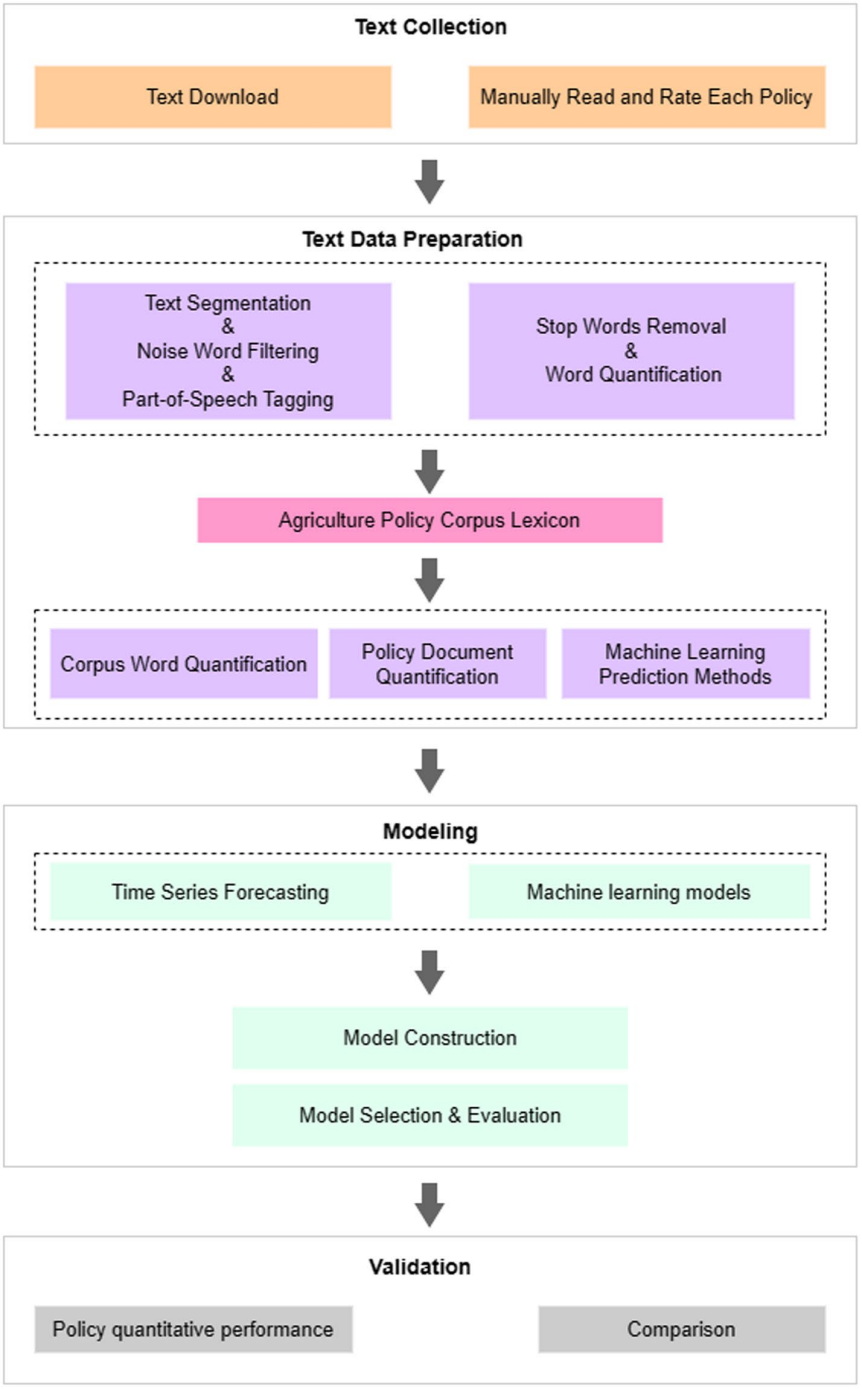
Research Framework.

The research employs a methodological framework, as illustrated in Fig. 1, which consists of four main stages.

1. Text Collection: The first step involves gathering and screening a dataset of Chinese agricultural policies. This is achieved by initially browsing relevant websites that publish Chinese agricultural policies, downloading policy documents, and subsequently manually reviewing and annotating them, eliminating any documents that are not pertinent to agricultural policies.
2. Text Data Preparation: The dataset is then subjected to various preprocessing steps, including text segmentation, noise word filtering, and part-of-speech tagging. Stop words removal, and Word quantification is conducted. Furthermore, a machine learning-based topic extraction model is applied to create a Chinese agricultural policy corpus. The terms in the corpus are quantified, forming the foundation for evaluating the strength of agricultural policies.
3. Modeling: Time-series machine learning models are utilized to model and predict policy strength, followed by an in-depth research analysis and evaluation of the prediction models.
4. Validation: To assess the reliability of the corpus and policy quantification approach established in this study, a comparison is made with China’s total agricultural output.

### Data collection

The data set for this study draws from multiple credible platforms and institutions, such as the content of China’s No. 1 Central Document on agricultural policies, which can be found at (http://www.lswz.gov.cn/html/xinwen/index.shtml). Also incorporated are policies and expert opinions issued by the Ministry of Agriculture and Rural Affairs of the People’s Republic of China, retrievable from (https://www.moa.gov.cn/), and materials from the Chinese Academy of Agricultural Sciences, accessible via (https://www.caas.cn/). The No. 1 Central Document encompasses a total of 25 agricultural policy documents spanning from 1982 to 2023, which have been identified through manual reading. The Ministry of Agriculture and Rural Affairs of China contributes a total of 93 policies, covering the period from 2010 to April 30, 2023. Furthermore, the Chinese Academy of Agricultural Sciences provides 310 expert opinions, spanning from 2011 to April 30, 2023. According to Table 1, following an extensive period of policy classification, a total of 428 documents pertaining to agricultural policies in China form the initial dataset for this study.

**Table 1:** Dataset of Chinese Agricultural Policies.

Category	Date	Quantity (article)	Total (article)
Document No. 1 of the Central Committee of China	1982–2023	25	428
Ministry of Agriculture and Rural Affairs of the People’s Republic of China	2010–2023	93	
Chinese Academy of Agricultural Sciences	2011–2023	310	

The term “Central Document No. 1” refers to the most significant policy document released annually by the Central Committee of the Communist Party of China. It is also commonly known as the “New Year’s Document” and represents a pivotal guiding document formulated by the central leadership for the new year. It is typically released at the beginning of each year and encompasses policy pronouncements and key work arrangements by the central government across various domains such as the economy, politics, and culture. Notably, since the onset of economic reforms and opening up, China has witnessed a widening gap between urban and rural economies, drawing increased attention to issues like agricultural modernization, rural income growth, and rural revitalization. Consequently, in most years, the Central Document No. 1 focuses on agricultural and rural issues, outlining targeted policy measures and work requirements. From 1982 to 2023, documents related to agricultural and rural work were issued in the years 1982 to 1986 and from 2004 to 2023. Consequently, through manual selection and annotation, we identified a total of 25 documents from the Central Document No. 1 during these years, which genuinely center on Chinese agricultural policy. Given that the specific details of agricultural policies proposed by other government departments are generally based on the Central Document No. 1, the corpus extraction is centered around these documents.

The Ministry of Agriculture and Rural Affairs of the People’s Republic of China is the highest agricultural authority in the country. It plays a crucial role in leading and managing China’s agricultural sector, rural economy, and rural social affairs. The ministry is tasked with developing and implementing the country’s agricultural and rural development strategies, formulating agricultural and rural policies, and promoting advancements in agricultural science and technology as well as modern agricultural practices. With its comprehensive responsibilities, the ministry plays a pivotal role in shaping and advancing China’s agricultural and rural sectors.

The Chinese Academy of Agricultural Sciences is a prominent agricultural scientific research institution within the People’s Republic of China. Its primary mission is to provide scientific and technological support and services to facilitate the country’s economic development and agricultural modernization. The academy conducts research in various fields of agricultural science and technology, agricultural resources and environment, and agricultural socio-economic aspects. Through its extensive research efforts, it plays a vital role in promoting the development of China’s agriculture.

In this study, we establish the China Agricultural Policy Corpus by examining the policy documents put forth by different agricultural government departments. These policies are developed in accordance with the requirements of the China Central Document No. 1, and they are organized for implementation and monitoring. China Central Document No. 1, as the core guiding document, serves as a standardized benchmark, enabling us to delve into the changing trends in the intensity of China’s agricultural policies. During the quantitative scoring phase, we comprehensively assess policy documents from the Ministry of Agriculture and Rural Affairs of China and the Chinese Academy of Agricultural Sciences. These assessments, combined with the analysis of the China Central Document No. 1, allow us to explore the trends in the intensity of agricultural policies in China from 1982 to April 30, 2023.

### Text preprocessing

To study the content of China’s agricultural policy, this research requires preprocessing the policy articles to extract keywords from the content, establish a corpus, and conduct quantitative policy analysis. The preprocessing stage involves several key steps, including text segmentation, noise word filtering, part-of-speech tagging, and stop words removal^16,17^.

1. Text segmentation:
  Text segmentation is essential for mining text data. It involves dividing continuous text sentences into separate words using specific rules and methods. However, Chinese has a complex semantic structure, and there is often a lack of clear word separators when using punctuation marks for sentence segmentation. This makes it challenging to filter out irrelevant words and identify words with practical meaning and value in Chinese agricultural policy texts. To address this challenge, the jieba library in Python programming can be utilized. Jieba is a widely-used Chinese text analysis tool known for its excellent segmentation capabilities^18,19^.
2. Noise word filtering:
  In the tokenized text matrix, encompassing all the vocabulary present in policy documents, not all words contribute significantly to the representation of policy intensity. Therefore, before forming a specific corpus, it is necessary to filter these words. Filter and remove words with frequencies lower than 0.001 and higher than 99.999, excluding words with excessively low or high occurrence rates. Additionally, delete non-substantive words such as personal names, place names, department names, etc. This process aims to refine the vocabulary by eliminating less relevant or potentially misleading terms, ensuring a more focused and meaningful representation of policy content.
3. Part-of-speech tagging:
  Part-of-speech tagging constitutes a crucial step in text preprocessing, serving to filter out irrelevant words while retaining essential keywords. In this study, we employed the jieba for part-of-speech tagging of the text matrix. Jieba is one of the most renowned toolkits in the field of natural language processing (NLP) and provides its own annotated Chinese language corpus. This allowed us to filter out nouns from the tagged results to create a noun dictionary.
4. Stop words removal:
  Removing stop words is a crucial step in data cleaning during the preprocessing stage. Stop words refer to common words that do not carry significant meaning in a given language, such as prepositions, articles, pronouns, and conjunctions^20^. By eliminating stop words during the extraction of the agricultural policy text corpus, the quality of the results can be improved, and meaningful vocabulary can be retained for analysis.
5. Word quantification:

*TF*-*IDF* (Term Frequency-Inverse Document Frequency) is a common text data preprocessing technology, which can convert text data into a vector representation to facilitate subsequent machine learning model training^21^. Among them, *Tf* (Term frequency) refers to the word frequency of a word in the corpus, and *Idf* (Inverse document frequency) refers to the inverse text frequency index in the corpus^22^. *Tf* is defined as shown in formula (1). Among them, *n*(*w*_*i*_) represents the number of occurrences of word *w*_*i*_ in the text matrix, and ${\sum }_{j=1}^{J}n\left({w}_{j}\right)$ represents the total number of occurrences of all words in the text matrix.

1 $$T{f}_{w}=\frac{n\left({w}_{i}\right)}{{\sum }_{j=1}^{J}n\left({w}_{j}\right)}$$

The inverse text frequency *Idf* is shown in formula (2), where *D* represents the total number of documents in the text matrix, and $n\left({t}_{i}\in {d}_{i}\right)$ represents the number of documents containing vocabulary *t*_*i*_ in the text matrix. Adding 1 to the denominator of the formula can prevent the denominator from being 0 and prevent the denominator from being 0 when the word *t*_*i*_ does not appear in all policy documents in the text matrix.

2 $$id{f}_{i}=\mathrm{log}\left(\frac{D}{n\,({t}_{i}\epsilon {d}_{i})+1}\right)$$

Through *Tf*_*w*_ and *idf*_*i*_, the *TF*-*idf* value can be calculated, as shown in formula (3). *TF*-*idf* is proportional to the number of times the word appears in the policy document and inversely proportional to the number of times the word appears in the entire text matrix. It is used for vector mapping and semantic feature extraction, which lays the foundation for the subsequent operation of the LDA algorithm.

3 $$TF-idf=Tf\ast idf$$

### Corpus construction

By analyzing agricultural policy texts, we can uncover underlying themes and identify words that more accurately capture their essence, enabling us to construct a comprehensive Chinese agricultural policy corpus. A policy document typically addresses various topics that can be manually distinguished, including policy background, goals, content, stakeholders, oversight, evaluation, communication, resources, and risks. Additionally, it often carries implicit topic-related information. To extract and analyze these topics and their associated lexical information, we leverage machine learning techniques such as topic modeling and visual analysis. Through the segmentation of each topic and the extraction of valuable lexical information, we create a Chinese agricultural policy corpus.

Topic extraction techniques involve analyzing and processing text using machine learning methods to extract multiple potential key topics from agricultural policies. These techniques present the vocabulary within each topic, obtaining lexical information under different themes in the field and forming the vocabulary of the Chinese agricultural policy corpus. There are various methods for mining text topics, with probabilistic graphical models being exemplified by Latent Dirichlet Allocation (LDA), and inductive reasoning-based methods represented by Nonnegative Matrix Factorization (NMF).

### EvoLDA-DB model

While the LDA model has shown effectiveness in extracting topics from agricultural policy texts, it encounters challenges when dealing with changes in text over time. LDA struggles to perform well in tasks involving temporal dynamics, and its assumption that each word in a document is influenced by multiple topics can lead to topic overlap. This overlapping may result in ambiguous topic word assignments, making it challenging to clearly allocate words to specific themes. To address the evolving dynamics of text over time, we have made improvements upon LDA, introducing a Time-Sliced Dynamic LDA model. Additionally, in the stage of topic word allocation, we have incorporated a density-based clustering algorithm, DBSCAN, to enhance the precision of topic assignments. This modification aims to overcome the limitations of LDA in capturing temporal variations and achieving more accurate topic delineations in dynamic text scenarios.

LDA is an unsupervised machine learning technique that utilizes a probabilistic graph model^23^. It determines and extracts words based on the contextual probability of words within documents and possesses certain empirical characteristics. Let’s assume the collection of documents is represented as D, the collection of topics as T, and the vocabulary in the documents as w. The relationship between topics, documents, and vocabulary in LDA can be expressed by formula (4), where p(w|d) denotes the probability of a word appearing in a document, p(t|d) represents the probability of a specific topic t appearing in the document, and p(w|t) indicates the probability of a particular word w appearing in the topic. Based on this formula, we observe that a topic t serves as an intermediary layer between documents and vocabulary. The relationship between topics and keywords can be derived by calculating the probability of each word in the document.

4 $$p\,(w| d)=p\,(w| t)\ast p\,(t| d)$$

The distribution of document-topic and topic-word follows a multinomial distribution, which can be represented by the Dirichlet distribution. The probability density function of the Dirichlet distribution is expressed as formula (5)^24^, where α represents the parameter of the multinomial distribution, and *p*_*k*_ represents the probability of the topic.

5 $$f(p{\rm{| }}a)=\left\{\begin{array}{ll}\frac{1}{\Delta (\alpha )}{\prod }_{k=1}^{k}{p}_{k}^{{\alpha }_{k}-1}, & {p}_{k}\in (0,1)\\ 0, & others\end{array}\right.$$

**Fig. 2:**
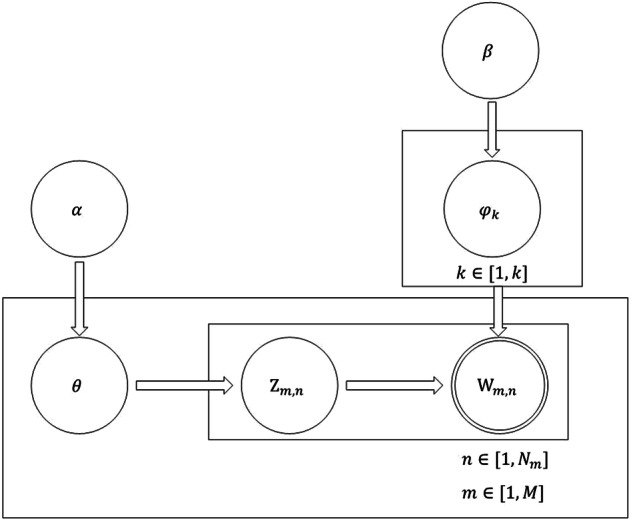
LDA model.

Let’s assume there are k topics in m documents. Each document has its topic distribution, which follows a Dirichlet distribution with a parameter *α*. Similarly, each topic has its topic-word distribution, which also follows a Dirichlet distribution with a parameter *β*. Each topic word in each document corresponds to a specific topic. The probabilistic graphical model representing these relationships is shown in Fig. 2^25^. We can use *θ* to represent the topic distribution. Thus, the topic of the *i*-th document denoted as *d*_*i*_, can be expressed as ${\theta }_{i}=\left({\theta }_{i1},{\theta }_{i2},\ldots ,{\theta }_{ik}\right)$. Additionally, *φ*_*k*_ represents the word distribution corresponding to the *k*-th topic, where $k\in \left[1,k\right]$. Hence, for the *k*-th topic, its topic-word distribution can be expressed as ${\varphi }_{k}=\left({\varphi }_{k1},{\varphi }_{k2},\ldots ,{\varphi }_{kj}\right)$. Here, *Z*_*m,n*_ represents the *n*-th topic in the *m*-th document. By selecting *φ*_*k*_ through *Z*_*m,n*_, we can obtain the corresponding observed value, denoted as *W*_*m,n*_.

The DBSCAN algorithm is a density-based spatial clustering algorithm^26^, which can identify clusters of arbitrary shapes in data with “noise” under certain density conditions. The relevant definitions are as follows:

*E*_*ps*_-neighborhood: For a given object *p*, the neighborhood with a radius of *E*_*ps*_ is called the *E*_*ps*_-neighborhood of that point. For any point *q* within the neighborhood of this point, it satisfies:

6 $$dist\left(p,q\right)\le {E}_{ps}$$

MinPts Threshold: The number of points within the *E*_*ps*_-neighborhood must be no less than the threshold MinPts, indicating the minimum set of points for a cluster.

Core Point: A point within the *E*_*ps*_-neighborhood of object p with a sample count exceeding the MinPts threshold.

Direct Density Reachable: If the object point *q* is within the neighborhood of the core point *p*, then *q* is considered directly density reachable from *p*.

Density Reachable: If there exists a chain of objects *p*_1_, *p*_2_, ……, *p*_*n*_, such that for any *p*_1_ to *p*_*i*+1_, they are directly density reachable, then *p*_0_ to *p*_n_ are considered density reachable.

Noise Point: Points outside the cluster formed by density reachable core point *p*.

Density Connected: In the sample set, when all objects are density reachable, they are considered density connected.

The DBSCAN clustering algorithm merges the largest datasets connected by density within the neighborhood of the sample set into a cluster. Subsequently, all groups of density-connected samples are assigned to different categories. The algorithm proceeds as follows:

Scan the entire dataset D and randomly select an unclassified object point *p*. Check if the number of density-reachable points within the *E*_*ps*_ neighborhood radius of point *p* is greater than the threshold MinPts. If it is, create a clustering cluster with *p* as the core data object. Iteratively cluster from this point, querying all density-reachable object points and forming a cluster. Points outside the cluster are labeled as noise. During the clustering process, it is possible to merge clusters that are density-reachable from core data points. Throughout the algorithm execution, some core points’ density-reachable objects may be queried repeatedly. After completing clustering for one cluster, proceed to select the next point for clustering until all data objects are assigned to a cluster or labeled as noise, concluding the clustering process.

**Fig. 3:**
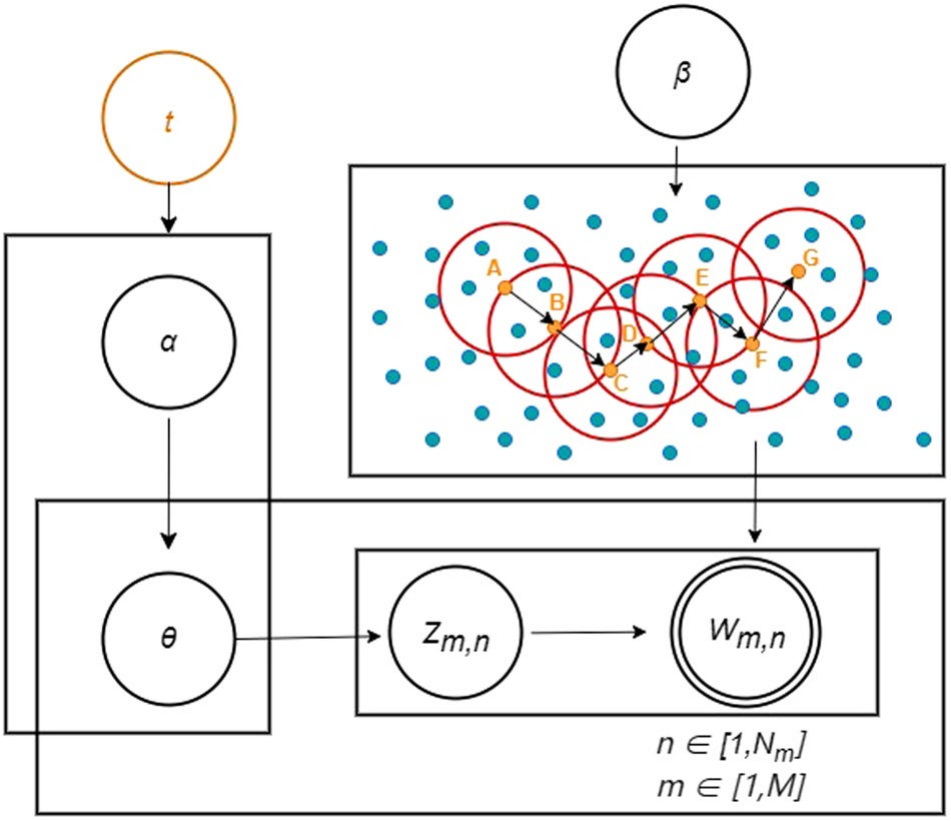
EvoLDA-DB model.

In this study, the primary focus is on the text of agricultural policies, divided by year. Relevant thematic keywords are extracted for each respective year. Semantic distances between these keywords are then calculated. By obtaining the probabilities of document-topic and topic-keyword relationships, a density-based noise-activated spatial clustering algorithm is applied to partition the thematic keywords. The EvoLDA-DB (Evolutionary LDA with DBSCAN) model, based on time-slice clustering, is illustrated in the Fig. 3.

The figure reveals that the EvoLDA-DB model extends the LDA model by incorporating a temporal component denoted as time slice “*t*.” Additionally, it replaces the word distribution *φ*_*k*_ corresponding to each topic with the word distribution obtained through the DBSCAN clustering algorithm. The parameters *α* and *θ* are considered mappings within a specific time slice “*t*” (in this study, a time slice corresponds to a year). The core data points (ABCDEFG) with density reachability are merged into clusters. During the algorithm execution, clustering proceeds by selecting the next point for clustering after completing clustering for a cluster. This process continues until all data objects are assigned to a cluster or labeled as noise, signifying the end of clustering. By partitioning time differently, the EvoLDA-DB model dynamically processes multiple textual data sets.

### Quantification of agricultural policy intensity

To investigate the changes in the intensity of China’s agricultural policies, it is essential to quantify the words within the corpus. This quantification serves as the basis for delineating the intensity of agricultural policy documents. During the word quantification stage, each word in the corpus exists within a specific topic and is distinct from others. Furthermore, words within the same topic convey similar meanings or belong to the same category. Consequently, the importance and quantitative value of each word within a topic are considered equivalent. In this study, we propose calculating the word frequency for each word in the original agricultural policy documents. By accumulating the word frequency values within each topic, we obtain a unified quantitative value for each topic. This quantitative value represents the quantitative value of words associated with each group of topics. Next, the strength of each agricultural policy document is quantified by combining the quantitative value of words with the frequency of occurrence of each word in the policy documents. This combined approach allows for the evaluation of the strength or intensity of agricultural policies, providing valuable insights into their future implications.

The word quantification of the agricultural policy corpus is determined by counting the word frequency (*Tf*) of words that appear in the original policy documents, as shown in the formula (1). Subsequently, the word frequency values of all words under each topic are summed to obtain the quantization value (*Tp*) for each topic, as illustrated in formula (7). The quantization value represents the strength of quantization for all words within a particular topic. Here, *j* represents the word order within a topic, while *Tf*_*j*_ represents the frequency value of the *j*-th word.

7 $$Tp={\sum }_{j=1}^{j}T{f}_{j}$$

To quantify the strength of each agricultural policy document, it is necessary to preprocess the content of the document by performing word segmentation. This process involves splitting the text into individual words. Next, the words in the agricultural policy corpus are matched with the word matrix derived from the segmented policy text. The frequency of each word in the agricultural policy corpus within the policy document is then counted. Subsequently, the quantitative value of each word in the corpus is multiplied by its corresponding statistical frequency in the policy document. These values are accumulated to obtain the quantitative intensity value of the agricultural policy document. The calculation method for the Agricultural Policy Intensity (*ARI*) is shown in formula (8). Here, *Tp*_*i*_ represents the quantitative value of the *i*-th word in the agricultural policy corpus, while *n*_*i*_ represents the frequency of the *i*-th word appearing in an agricultural policy document.

8 $$ARI={\sum }_{i=1}^{i}T{p}_{i}\ast {n}_{i}$$

### Machine learning prediction methods

In this study, two main approaches are employed. Firstly, a machine learning method is utilized to construct a corpus that is specifically tailored to China’s agricultural policy. Secondly, the relationship between the constructed corpus and policy strength is examined through the application of text analysis and quantitative strength modeling techniques.

Given the specific characteristics of China’s agricultural policy documents, six groups of algorithm models suitable for time series analysis and forecasting are selected for evaluating the strength of China’s agricultural policies. These models include Prophet, autoregressive moving average model (ARIMA), recurrent neural network (RNN), gated recurrent unit (GRU), long short-term memory network (LSTM), and bidirectional long-term short-term memory network (Bi-LSTM).

Both Prophet and ARIMA are statistical models that leverage the statistical characteristics of time series data for modeling and forecasting purposes. These models are adept at capturing the trend, seasonality, and periodicity within time series data. Additionally, they offer strong interpretability and can provide statistical significance and confidence intervals for the model’s various parameters. On the other hand, RNN, GRU, LSTM, and Bi-LSTM are all neural network models specifically designed to handle sequence data, including time series data. These models excel at capturing contextual relationships and long-term dependencies within sequences by facilitating information transfer between different time steps. Notably, GRU, LSTM, and Bi-LSTM demonstrate exceptional performance when dealing with long-term dependencies. They can effectively retain and update past information, thereby enhancing the accuracy of future value predictions. Given the impressive performance of these selected algorithms in various domains, they can effectively support the objectives of this research.

To scientifically evaluate the performance of the chosen prediction model in this study, we employed commonly used performance measurement standards to analyze the performance of each specific task. Examples of such standards include the mean absolute error (MAE) and the root mean square error (RMSE), as depicted in formulas (9) and (10), respectively.

9 $$MAE=\frac{1}{n}{\sum }_{i=1}^{n}\left|\widehat{{y}_{i}}-{y}_{i}\right|$$

10 $$RMSE=\sqrt{\frac{1}{n}{\sum }_{i=1}^{n}{\left(\widehat{{y}_{i}}-{y}_{i}\right)}^{2}}$$

## Data Records

The research dataset comprises a total of 428 articles focusing on China’s agricultural policies spanning the period from 1982 to 2023, such as the content of China’s No. 1 Central Document on agricultural policies, which can be found at (http://www.lswz.gov.cn/html/xinwen/index.shtml). Also incorporated are policies and expert opinions issued by the Ministry of Agriculture and Rural Affairs of the People’s Republic of China, retrievable from (https://www.moa.gov.cn/), and materials from the Chinese Academy of Agricultural Sciences, accessible via (https://www.caas.cn/). These articles have been categorized and collected based on different government departments. Additionally, the dataset includes the Chinese agricultural policy corpus, the corresponding corpus word quantification scores, and the quantitative agricultural policy intensity scores obtained through machine learning model training. All data records have been uploaded to public data repositories, not only on GitHub (accessible at https://github.com/YNAU-WYH/Agricultural-policy-dataset.git), but also to figshare^27^ and the Science Data Bank^28^. These three repositories house identical content, ensuring comprehensive accessibility. The repository includes the following components:

A dataset containing the original text of China’s agricultural policy documents from 1982 to April 2023.

The Chinese agricultural policy corpus dictionary, along with its associated dataset of quantitative values.

A dataset presenting the agricultural policy intensity in China across the years 1982 to April 2023.

### Agricultural policy corpus

**Fig. 4:**
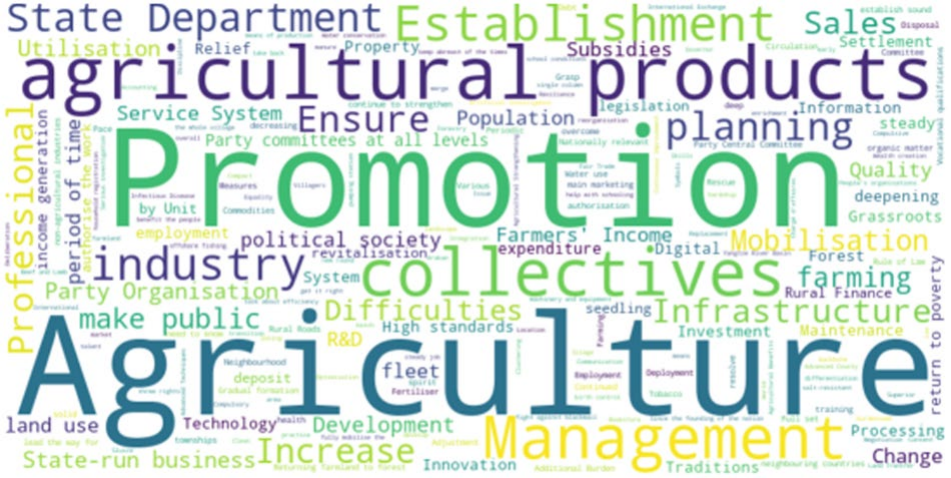
Word cloud diagram of the corpus.

This dataset provides 25 topics obtained through machine learning algorithm models, containing a total of 250 words from the Chinese Agricultural Policy Corpus, which are classified into 14 clustering dimensions, and according to each dimension, the quantitative values of the words under each dimension are provided by using the Agricultural Policy Corpus word quantification method. Specifically, the dataset encompasses the Chinese Agricultural Policy Corpus, which serves as the focal point of China’s Central No. 1 Document spanning from 1982 to April 2023. In Fig. 4, the word cloud diagram depicts word importance, where darker colors and larger fonts indicate higher importance, and vice versa.

To facilitate a better understanding and broader utilization of our agricultural policy corpus dataset, we have translated it into both Chinese and English before uploading it to the public data repository, GitHub. This translation allows researchers to effectively comprehend and reuse the dataset.

### Agricultural policy score sheet

The dataset encompasses 428 agricultural policy documents publicly released in China between 1982 and April 2023. Each document is accompanied by a corresponding intensity score table, which exhibits distinct characteristics reflecting the Chinese context. To facilitate researchers’ usage, we have classified the dataset based on different government agencies, resulting in three separate score tables.

**Fig. 5:**
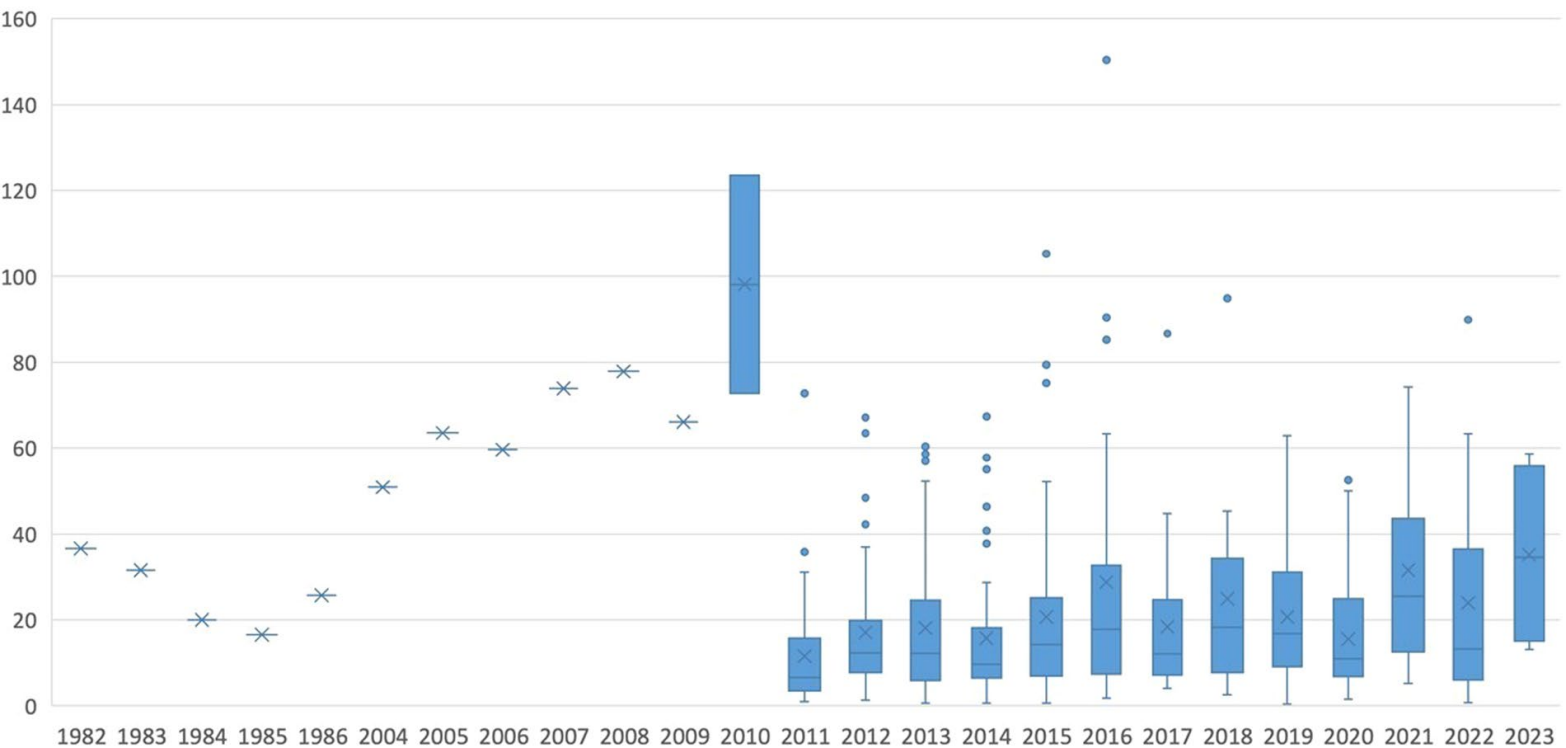
Box plot of China’s agricultural policy intensity distribution.

The dataset is presented in tabular form, containing the titles of agricultural policy documents in both Chinese and English, along with their corresponding scoring scores. To illustrate the distribution of the intensity of Chinese agricultural policies, we have created scatterplot interval boxplots for the policy intensity of each document over the years (see Fig. 5). This graph displays the quantity of agricultural policies in the dataset and their distribution trends in different years. The blue rectangular region represents the middle 50% of the data, the horizontal line represents the median, the upper dashed line contains 25% of high-value data, the lower dashed line contains 25% of low-value data, asterisks indicate outliers beyond the margins, and dots represent anomalies.

The number and intensity values of agricultural policies in China show a growing trend, especially over the past fourteen years. However, this growth trend is gradually slowing down. The intensity of agricultural policies fluctuated significantly in earlier years, especially reaching a higher level in 2010. Through analysis of the dataset, we found that this is because, during this period, the Chinese government significantly increased the number and intensity of agricultural policy releases compared to previous years. However, over time, the volatility in recent years has gradually decreased. Looking at the average trend of agricultural policy intensity, it has been on the rise in recent years. This reflects the continuous efforts of various government departments in China to strengthen regulatory measures and improve agricultural support policies in addressing agricultural issues.

## Technical Validation

### Collection of datasets

In this study, the dataset of agricultural policy documents spanning from 1982 to April 2023 was compiled by gathering agricultural policy documents issued by the Chinese government. The official websites of various government departments were used as the source of these documents. Rigorous and meticulous procedures were followed to ensure the accuracy and reliability of the dataset. The policy texts were thoroughly read, and multiple rounds of manual screening and marking were conducted to extract policies specifically related to agriculture. These measures were taken to ensure the precision and meticulousness of the data source.

### Inspection of agricultural policy corpus construction

After collecting the publicly available agricultural policy dataset from the Chinese government, a series of preprocessing steps were applied to the data, including segmentation, noise word filtering, part-of-speech tagging, stop-word removal, and word quantification. In the noise filtering stage, considering the characteristics of the dataset from China’s Central Document No.1, several sets of parameters were selected for comparison, as shown in Table 2. Among them, the best quality of retained words was achieved by filtering out noise words with a frequency lower than 0.001 and higher than 99.999.

**Table 2:** Noise word filtering parameters.

Word frequency reservation range	Average number of words per year (pcs)
0.1∼99.9	28.28
0.01∼99.99	476.68
0.001∼99.999	1574.68
100	1877.16

On the basis of the Latent Dirichlet Allocation (LDA) model, we introduce, for the first time, the temporal dimension and the clustering algorithm DBSCAN to partition topics. We further enhance this approach, naming the newly proposed machine learning model EvoLDA-DB (Evolutionary LDA with DBSCAN), to construct an agricultural policy corpus. In addition to utilizing DBSCAN for topic partitioning, we also experiment with the Canopy clustering algorithm.

In 1999, Lee and Seung proposed a Nonnegative Matrix Factorization algorithm (NMF) in Nature^29^, abbreviated as NMF. It maps text or data into a model that computers can measure, and then categorizes vocabulary through word distance calculations. NMF is effective in extracting latent information from short texts. Following a similar improvement approach as LDA, we optimize NMF and introduce Topic Coherence as an evaluation metric for testing the quality of topics, conducting a model comparison.

There exists a certain relationship between the number of topics and the quality of topics. As the number of topics increases, the quality of a specific topic gradually deteriorates. This is manifested in a reduction of the association between topic words and the topic, leading to an inability of the topic words to accurately reflect the exact meaning of the topic^30^. Therefore, Topic Coherence serves as a reliable measure for assessing the quality of topic words.

Let *D*_(*v*)_ represent the frequency of word *v* in document *D*, and *D*_(*v,v*′)_ denote the probability of both words *v* and *v*′ appearing together in document *D*. The Topic Coherence of document topics can be expressed as:

11 $$C\left(t,{V}^{\left(t\right)}\right)={\sum }_{m=2}^{M}{\sum }_{l=1}^{m-1}log\frac{D\left({V}_{m}^{\left(t\right)},{V}_{l}^{\left(t\right)}\right)}{D\left({V}_{l}^{\left(t\right)}\right)}$$

The list *V*^(*i*)^ represents the top m words with high probabilities in a given topic. As observed from Eq. 11, the numerator corresponds to the frequency (or probability) of co-occurrence of two words, while the denominator represents the frequency (or probability) of occurrence of either of the words. The higher the semantic relevance between two words in a specific topic, the more likely they are to belong to the same theme, indicating a higher consistency of topic words.

As shown in Table 3, using PCA as the baseline, we calculate the Topic Coherence values for different topic models, including NMF, LDA, EvoNMF-Canopy, EvoLDA-Canopy, EvoNMF-DB, and EvoLDA-DB. It can be observed that EvoLDA-DB achieves the highest Topic Coherence value. Therefore, we choose the EvoLDA-DB model to extract the Chinese Agricultural Policy Corpus.

**Table 3:** Evaluation of Topic Coherence for Agricultural Policy Subject Headings.

Model	Topic Coherence
PCA	0.50343933
NMF	0.487888867
LDA	0.623404476
NMF-Canopy	0.746787886
LDA-Canopy	0.576346577
NMF-DB	0.971816266
EvoLDA-DB	0.979527622

For the extraction of the Chinese Agricultural Policy Corpus using the machine learning algorithm EvoLDA-DB, considering the dataset containing 25 policies from China’s Central Document No. 1, we set the number of topics (K) to 25 during the training of the EvoLDA-DB algorithm. The hyperparameters alpha and beta are set to 0.2 and 0.01, respectively. The number of iterations (num_iters) is set to 1000 rounds, as indicated in Table 4.

**Table 4:** Hyperparameters of EvoLDA-DB model training.

Hyperparameters	value
K	25
alpha	0.2
beta	0.01
num_iters	1000

### Agricultural policy strength test

Considering the quantification of words in the corpus, there is the problem of a very small number of words that may be of high importance but may occur less frequently in policy documents. We found that such words with low frequency but high importance are often surrounded by rich substantive passages that elaborate and specify the meaning of the agricultural policies covered by these words, so that even if these keywords do not appear much, the essence of the information they carry has been integrated into the whole text through the associated descriptive expressions, which can be covered by the corpus words constructed in this study. Therefore, the policy content referred to by these low-frequency and high-weight words has been effectively captured and analysed. Secondly, when the words with very low frequency but high importance appear, in the policy documents issued throughout the year, the documents with this theme or core will appear with high frequency, resulting in the result that the meaning of the agricultural policy involved in the elaboration and concretisation of these words accompanied by these words will be increased in a large scale, and the policy content referred to by these low-frequency and high-weight words will be included in the corpus created in this study.

Agricultural output value serves as a core indicator reflecting the level of agricultural development in a country or a specific region, whereas policy intensity embodies the extent of government support and intervention in the agricultural sector. Robust agricultural policies are typically accompanied by a suite of measures, including higher fiscal investments, technological innovation promotion, and structural optimization of the industry, all of which directly influence agricultural production efficiency and value-added growth. Consequently, there exists an inherent logical connection between policy intensity and agricultural output value, suggesting that a higher policy intensity is expected to contribute favorably to increasing agricultural output. Policy documents, once released, undergo a series of stages, including publicity, planning, implementation, execution, and ultimately, exerting their effects across various aspects of agricultural production. Agriculture, being a long-term investment process with subsequent yields, generally experiences a time lag between policy formulation, its actual implementation, and the realization of economic benefits.

To validate the scientific nature of the method employed in this study, the Chinese agricultural policy corpus spanning over 40 years from 1982 to 2023 was analyzed using machine learning techniques. This analysis involved quantifying the value of each word and constructing a score for agricultural policy intensity. To further assess the validity of the method, the study incorporates data on China’s total agricultural output value sourced from the “China Statistical Yearbook” compiled by the National Bureau of Statistics of China. To examine the observed correlation between policy intensity and agricultural output, the study utilizes the discrete correlation function (DCF). This statistical method allows for an investigation of the relationship and potential correlations between the intensity of agricultural policies and the corresponding agricultural output. By applying the DCF, the study aims to assess the degree of correlation and establish the scientific basis of the method employed.

The “China Statistical Yearbook” is an annual comprehensive statistical reference book published by the National Bureau of Statistics of China. It provides detailed statistical data and information across various fields in China. The primary purpose of the Statistical Yearbook is to present the state of China’s national economic and social development. It serves as a valuable reference for government decision-making, academic research, and the general public.

The discrete correlation function (DCF) method was originally proposed by Edelson and Krolik in 1988^31^, and later improved upon by Hufnagel and Bregman in 1992^32^ who introduced a more accurate error estimate and extended the method. DCF is particularly suitable for analyzing non-uniformly sampled data and is commonly employed to identify correlations and time delays between two datasets. The fundamental concept behind DCF involves grouping the two columns of data based on their time differences. These time differences, referred to as delay times or lags, are then utilized to calculate the correlation function for each specific group^33^. When computing the discrete correlation function, it is essential to calculate the correlation function of the undivided “*bin*” first, known as the unbinned correlation function (*UDCF*). This UDCF is determined using formula (12) and serves as a key component in the overall calculation process.

12 $$UDC{F}_{ij}=\frac{\left({x}_{i}-\bar{x}\right)\left({z}_{j}-\bar{z}\right)}{{\sigma }_{x}{\sigma }_{z}}$$

13 $$\Delta {t}_{ij}={t}_{j}-{t}_{i}$$

When the error has a significant impact on the data, it is necessary to modify the denominator of the aforementioned formula to $\sqrt{\left({\sigma }_{x}^{2}-{\in }_{x}^{2}\right)\left({\sigma }_{y}^{2}-{\in }_{y}^{2}\right)}$. In this study, the influence of the error cannot be overlooked, and therefore the formula that includes the error term is utilized. Here, *σ*_*x*_ and *σ*_*y*_ represent the standard deviations of the two columns of data, while *t*_*i*_ and *t*_*j*_ correspond to the respective time points of the two datasets. To perform the DCF calculation, the data is grouped based on the time differences falling within the interval $\tau -\Delta \tau /2\le \Delta {t}_{ij} < \tau +\Delta \tau /2$. Each group contains *M*(*τ*) data points, where τ represents the time delay (*lags*). The calculation of the *DCF* is depicted in formula (14), and the variance (*Var*) is given by formula (15).

14 $$DCF\left(\tau \right)=\frac{1}{M\left(\tau \right)}{\sum }_{k=1}^{M\left(\tau \right)}UDC{F}_{ij}$$

15 $$Var\left(\tau \right)=\frac{1}{{\left(M\left(\tau \right)-1\right)}^{2}}{\sum }_{k=1}^{M\left(\tau \right)}{\left[UDC{F}_{ij}-DCF\left(\tau \right)\right]}^{2}$$

**Fig. 6:**
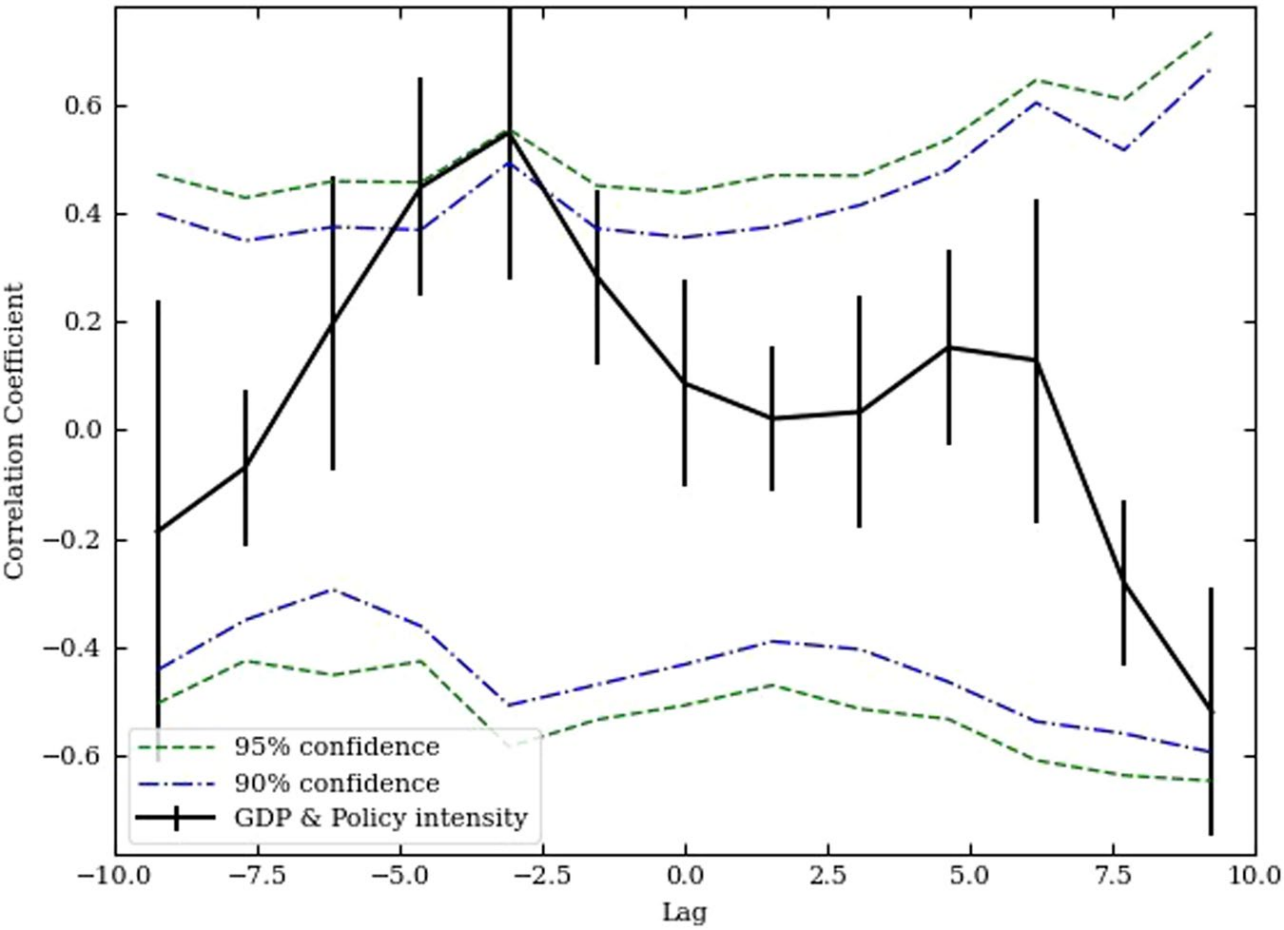
The result of DCF.

The agricultural total output data from the China Statistical Yearbook for the years 2005 to 2021 were collected for this study. The policy intensity, as per the corresponding year’s Central Document, was calculated using the Discounted Cumulative Flow (DCF) method. Table 5 presents the values of the relevant hyperparameters, lag intervals, and step sizes after fine-tuning. The computed results are illustrated in Fig. 6, where the blue and green lines represent the 90% and 95% confidence intervals, respectively^34^. From the DCF results, it is evident that the correlation coefficient between China’s agricultural total output and the agricultural policy intensity lags by 2.5 years, showing the highest positive correlation. Consequently, the period from the issuance of agricultural policies in China to their impact on total agricultural output spans a duration of 2.5 years. This underscores the significant influence of China’s agricultural policy proclamation on the total agricultural output, validating the scientific and practical viability of the methodology employed in this study.

**Table 5:** Hyperparameters of DCF.

Hyperparameters	Value
Lag Lower Bound	−10
Lag Upper Bound	10
Step Size	0.25

### Model prediction

In this study, we conducted training and testing of a machine learning predictive model to estimate the intensity of China’s agricultural policy from 1982 to 2023. Six sets of algorithm models were employed, namely Prophet, ARIMA, RNN, GRU, LSTM, and Bi-LSTM.

To evaluate the models during training, the agricultural policy datasets were randomly split into a training set (80%) and a test set (20%). Each group of machine learning prediction models was applied to the training set, and the trained models were then applied to the test set to evaluate the test error. The results are summarized in Table 6.

**Table 6:** Performance of the prediction model.

Model	MAE	RMSE
Prophet	15.62	20.35
Arima	23.85	32.21
RNN	12.44	18.74
GRU	18.18	21.94
LSTM	12.83	19.51
Bi-LSTM	12.55	18.50

**Fig. 7:**
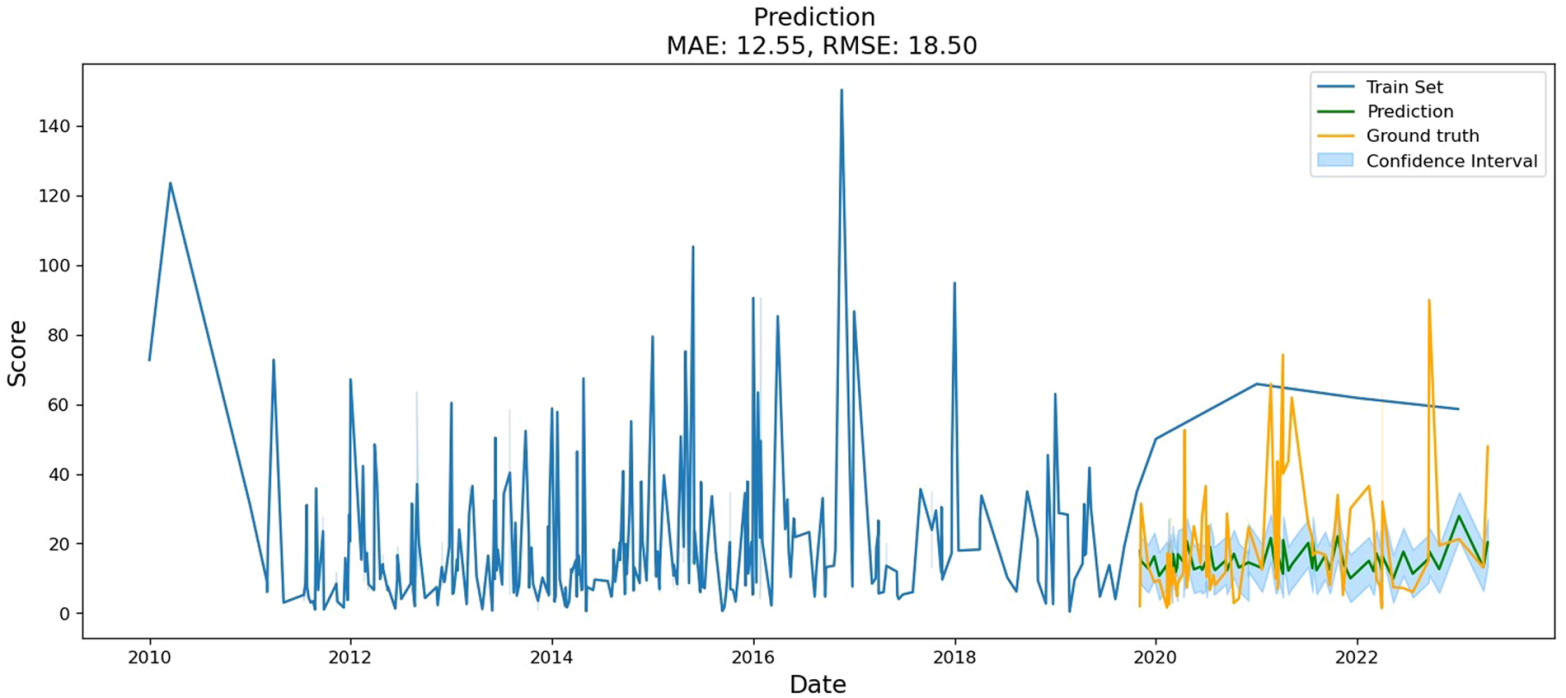
Prediction map of China’s agricultural policy intensity based on the Bi-LSTM model.

As shown in Table 5, the bidirectional long-term short-term memory network (Bi-LSTM) achieved the lowest root mean square error (RMSE) among all models, indicating the best performance. Therefore, Bi-LSTM was selected as the model to measure the strength of China’s agricultural policy. Using the Bi-LSTM model after 100 training rounds, a forecast map of China’s agricultural policy intensity was generated (as depicted in Fig. 7). The forecast graph clearly illustrates the trend of the green forecast curve, which performs favorably when compared to the actual data curve. Additionally, a confidence interval, based on a 95% confidence level, is provided. This confidence interval demonstrates the credibility of the prediction results and offers a more reliable solution for the subsequent prediction of China’s agricultural policy intensity.

### Data set reusability

This study introduces a novel method and approaches to constructing a comprehensive Chinese agricultural policy dataset, including the creation of a Chinese agricultural policy corpus, a quantitative corpus, and the measurement of the strength of China’s agricultural policy.

To construct the Chinese agricultural policy corpus, the study collects agricultural policy documents released by the Chinese government. After performing data preprocessing, a machine learning unsupervised algorithm called the EvoLDA-DB model is utilized to extract subject terms from these agricultural policy documents spanning the period from 1982 to 2023. By fine-tuning the hyperparameters of the training model, a set of 250 unique corpus words is obtained to build the Chinese agricultural policy corpus.

To quantify the strength of China’s agricultural policies, the study proposes a method that utilizes word frequencies within the agricultural policy corpus to quantify the importance of words and measure the strength of agricultural policy documents by counting their frequencies within policy documents. This method is validated by examining the collected agricultural output value and policy intensity data from the China Statistical Yearbook spanning from 2005 to 2021. The study reveals a strong positive correlation between the two variables with a delay of 2.5 years, thereby verifying the feasibility and accuracy of the quantitative method employed.

Furthermore, by observing the average trend of China’s agricultural policy intensity from 1982 to 2023, the study identifies an upward trajectory in recent years. This trend reflects China’s continuous efforts to enhance the supervision of agricultural issues through various types of agricultural policy measures.

The study employs a machine learning forecasting model to conduct a detailed analysis and forecast of China’s agricultural policy intensity from 1982 to 2023. Through the comparison of multiple prediction models, the study identifies the Bi-LSTM model as the most accurate, with a prediction root mean square error (RMSE) of 18.5. Thus, the Bi-LSTM model of machine learning provides an accurate prediction and deep analysis of the future intensity of China’s agricultural policy.

Overall, this study presents a comprehensive and robust approach that can assist decision-makers and policy makers in better understanding and addressing future agricultural policy challenges. Additionally, the datasets generated in this study can serve as valuable resources for future research endeavors in related areas.

## Usage Notes

The study demonstrates the collection of reliable data from multiple sources, including the content of China’s Central No. 1 Document on agricultural policies, and expert opinions from the Ministry of Agriculture and Rural Affairs of China, and the Chinese Academy of Agricultural Sciences. Based on this data, the study systematically constructs the Chinese agricultural policy corpus and proposes a quantification method to evaluate the strength of each agricultural policy document. The analysis and verification of these data provide valuable insights into the validity of the Chinese agricultural policy corpus and the evaluation results of policy strength.

The research results have important practical significance and lay the foundation for future studies in various aspects.

Firstly, the establishment of the Chinese Agricultural Policy Corpus dataset is highly significant for its application in the agricultural field. Researchers can benefit from this corpus by overcoming the challenges associated with exploring China’s agricultural policies. Additionally, the quantification method proposed for policy strength not only allows for comparisons between countries but can also be applied to different fields.

Secondly, while the dataset resulting from the quantification of the Chinese Agricultural Policy Corpus provides valuable insights, it is important to note that the quantitative value of policy intensity is only the initial step in evaluating agricultural policies. The ultimate impact of these policies depends on their implementation. Future research could explore regulatory actions in policy implementation as measures of the magnitude of the action and their impact on agricultural output. By comparing the intensity and enforcement of agricultural policies, key factors for the successful regulation of agricultural policies can be identified.

Lastly, this study focuses on the content of China’s agricultural policies, which are usually further refined and implemented by Chinese provinces and local governments with some adjustments. Therefore, future research can apply the constructed method of agricultural policy intensity to evaluate the implementation intensity of agricultural policies in various provinces or local governments in China. Furthermore, by employing the policy quantification method used in this study, additional datasets from various provinces or local governments can be collected to obtain a more comprehensive indicator of policy intensity. Comparing this intensity with the GDP of each province can help explore the relationship between the two factors.

## Data Availability

The code data utilized for computations and analyses within this paper is accessible via the GitHub repository at (https://github.com/YNAU-WYH/Agricultural-policy-dataset.git).
